# Demographics, Practising Arrangements, and Standards: Survey among New Zealand Dentists

**DOI:** 10.1155/2018/7675917

**Published:** 2018-11-18

**Authors:** Robert James Lee, Jithendra Ratnayake, Arthi Veerasamy, Carolina Loch, Peter Cathro, Paul A. Brunton

**Affiliations:** University of Otago, Faculty of Dentistry, 310 Great King Street, Dunedin 9016, New Zealand

## Abstract

**Background:**

To determine the demographic profile and practising arrangements of general dentists in New Zealand.

**Methods:**

A questionnaire comprising 19 sections with 125 questions was distributed via mail to 351 general dentists in New Zealand who were selected, at random, from the Dental Council of New Zealand's 2016 register.

**Results:**

Two hundred and four questionnaires were returned, of which 188 were usable giving a response rate of 53.5%. The majority of the respondents (63.5%) were male and practice principals (56.8%). Fifty-nine percent of the practices were located in city or town centres with a wide geographic distribution. Sole practitioners accounted for 24.1% of respondents, with the mean number of dentists per practice being 3.2. The majority of respondents (71.6%) attended five or more continuing professional development courses in the past year. Ninety-one percent of respondents used a computerized management system, and 95.3% used the Internet. The use of nickel-titanium endodontic files (83.9%) and digital imaging (82.2%) was the most frequently cited clinical innovations. Articaine was the most popular local anaesthetic of choice.

**Conclusions:**

Dentistry is an ever-changing profession, with evidence that NZ dentists continue to develop, learn, and embrace advancements in technologies to supply high-quality evidence-based treatment.

## 1. Introduction

Patterns of dental disease have changed dramatically over the past quarter century. Public awareness of the benefits of good oral health has increased, and the impact of advances in personal and public prevention strategies, including fluoride toothpaste and fluoridated water supplies, is now being realised. Although the amount of simple treatment provided is declining, the amount of complex treatment required and provided is correspondingly increasing [1]. With the increase in average life expectancy, adults are retaining more of their natural dentition for longer, guiding the strategic vision for oral health to be focused on “good oral health, for all, for life” [2, 3]. The changing oral health needs of the population highlight the task at hand for the dental profession in treating dental disease and maintaining oral health and quality of life for patients. In addition, patients now have a greater awareness of dental aesthetics and expect to remain dentate throughout life.

Dentistry is a commercialised profession, which is reflected in a number of ways such as the growing number of postgraduate courses, business aspects of dentistry, and marketing of alternative forms of treatment and materials. With the proliferation of digital technology over the past ten years, a new “communication culture” has also emerged. Due to the attractiveness and popularity of social media, many dental schools also use social media to promote their courses and communicate with their students [4]. In addition, social media have been used to promote new dental technologies and advertise private dental practices, as well as to share dental research [5]. The degree to which social media is used by general dentists is an area that has to date been poorly investigated.

Questionnaire surveys provide a valuable insight into the practising habits of general dentists. This allows the gathering of baseline data against which future studies can be compared. Similar studies have been conducted in the United Kingdom to investigate the practising arrangements, use of materials, techniques, and related technologies in dental practice [5–7]. These studies provided valuable information regarding trends in primary dental care, new technologies adopted by dentists, and the increasing commercialisation of dentistry. In addition to this, studies of this kind allow for investigation of changes occurring within the profession as general dentistry adapts to the ever-changing driving forces centred in patients' high dental need and continuous developments in new technologies, equipment, and procedures.

With this in mind, the purpose of this three-part series is to present and discuss the findings of a study investigating the practising arrangements and habits of general dentists in New Zealand, including new data on Internet and social media usage for patient communication. The subsequent papers in this series will report findings in relation to materials and techniques used for direct and indirect restorations, bleaching, endodontics, and paediatric dentistry.

## 2. Methods

Ethical approval was obtained from the University of Otago Human Ethics committee (approval number D16/098). A cross-sectional survey was conducted among general dentists practising in New Zealand who were holding a current annual practising certificate (APC) from the Dental Council of New Zealand in 2016. From 2131 dentists registered, contact details (e-mail addresses, postal addresses, telephone, and fax numbers) were available for only 1579 of them. From those, a subsample of 351 general dentists was selected at random from the register. Sampling was done proportionally to the number of registered dentists in each of the 14 NZ regions. A covering letter, paper copy of the questionnaire, consent form, envelope to return the completed survey form, and $5 coffee voucher were distributed in 2016 to the selected participants. The Questionnaire used in this study was previously validated in a similar UK-based study [5, 8]. The current questionnaire was modified to suit New Zealand dental practitioners and was piloted, prior to distribution amongst 10 New Zealand dental practitioners. The questionnaire comprised of 19 sections and 125 questions (the questionnaire is available on request from the corresponding author). Questions were based on those used in an annual survey of dentists in the USA by the Clinical Research Associates and also in a similar UK-based study [8]. It covered a variety of topics, including general practising information, preventive dentistry, restorative/operative dentistry, and paediatric dentistry. Exclusion criteria for this study included general dentists working in universities and government departments, retired from clinical practice, and general dentists who did not hold a current practising certificate.

An e-mail reminder was sent four weeks after the questionnaire was sent to all the nonrespondents. Data were analysed using Statistical Package for Social Studies software (SPSS version 24; IBM Corporation, Armonk, NY, USA). Summary statistics (mean and standard deviations) were presented as appropriate for each question. Cross tabulations and chi-squared tests were used to assess the statistical association between a number of demographic variables and other questions of interest. The level of significance was set at *p* < 0.05.

## 3. Results

### 3.1. General Demographic Data

Responses were received from 14 New Zealand regions and major cities. Due to the unequal distribution of the New Zealand dental workforce throughout the country, the responses received were uneven between the regions. Hence, the data from the returned questionnaires were weighted to correct for the potential survey bias (Table 1). The results for each table are reported as the actual frequency of the response and weighted percentage.

From 351 questionnaires sent, a total of 204 were returned, which represents a response rate of 58%. After checking the validity and completeness of returned questionnaires and applying the exclusion criteria, the final number of participants was 188.

Of the respondents, 63.5% (*n*=121) were male, 57% (*n*=107) were practice principals, and 37.5% (*n*=69) were associates. The majority of respondents (76%, *n*=141) worked in partnership/group practices, with the remaining were sole practitioners (*n*=47, 24%). A greater number of practices were found to be in city/town centres (*n*=114, 60%), followed by 30% (*n*=52) being suburban and 10% (*n*=22) in rural areas. The mean number of dentists per practice was found to be 3.2 (SD 1.99). The mean number of hygienists per practice was 1 (SD 0.97), and the mean number of therapists was 0.2 (SD 0.48). The mean number of qualified dental assistants per practice was 2.73 (SD 2.24), and the number of unqualified dental assistants was 1.36 (SD 1.60). The mean number of years since graduation was 23.6 years (SD 12.70).

### 3.2. Practice Workload

For the purpose of this study, a patient-care session was specified as a half-day. A mean of 17 (SD 14.50) dentist-patient care sessions were delivered per practice while a mean of 3.2 dentists (SD 1.99) worked in each practice. The number of sessions ranged between 1 and 80 depending on the practice location and number of dentists.

The mean number of therapist-delivered sessions per week was 1 (SD 0.48), and the mean number of hygienist-delivered sessions per week was 6 (SD 0.97). Hygienists typically treated 6 patients per session (SD 11.55), while therapists treated 1 patient per session (SD 3.41).  Table 2 shows the average availability of appointments for nonurgent care.

### 3.3. Method of Patient Payment

The proportion of patients treated under various arrangements such as private, accident compensation corporation (ACC), insurance, and dental benefit scheme was investigated. The respondents highlighted that the majority of patients are treated on a private basis (82%), 9% are treated under the dental benefits system, 5% under ACC, 2% via insurance, and 2% via other means, which includes funds from Work and Income New Zealand (WINZ) and funding through the hospital system.

### 3.4. Postgraduate Education

Over 71% of dentists attended five or more continuing professional development (CPD) courses over the past year. Twenty percent attended between three and four courses, 7% attended between one and two, and 2% did not answer this question.

**Figure 1:**
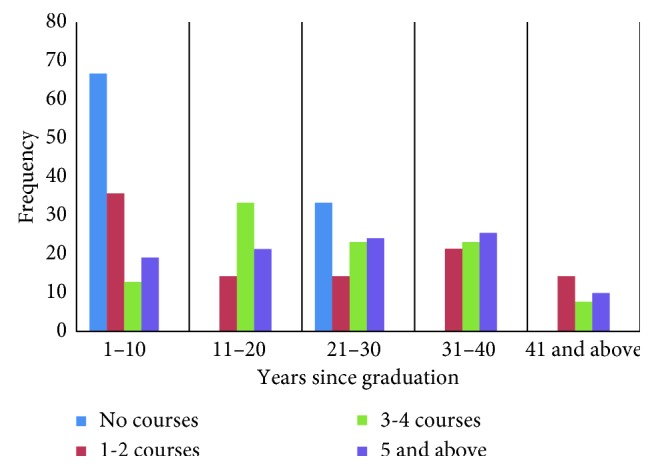
Association between the years since graduation and number of CPD courses attended.

There was no significant association between the time since dentists had graduated and the number of courses that they attended in the past year (*X*^2^ = 11.585, *p* < 0.05) (Figure 1). In addition to this, there was no significant association found between location of practice and number of courses attended (*X*^2^ = 4.650, *p* < 0.05).

### 3.5. Pain and Anxiety Control

When questioned on the methods of pain and anxiety control used in practices, local anaesthesia was the most common technique employed (used by 95% of practitioners). Regarding sedation, 24% of respondents used intravenous, 23% used nitrous oxide, and 20% used oral sedation. Seven percent of respondents referred patients for hospital or community-based general anaesthesia. There were no respondents who reported using acupuncture as a form of pain or anxiety control. However, there was one who reported using hypnosis. Articaine was the most popular type of local anaesthesia used (77%), followed by mepivacaine (41%) and lidocaine (36%).

### 3.6. Use of Practice-Based Computers

Responses showed that 91% of dentists used a computerized patient management system. The most commonly used system was *Exact* (Software of Excellence International, Auckland), which was used by 67% of participants. A total of 10% of respondents did not answer this question.

Of all respondents, 95% had access to the Internet and 95% used e-mails, mainly for correspondence (96%), making appointments (61%), ordering materials (60%), and other uses (8%) such as e-mailing radiographs, sending photos to the laboratory and referrals. Eighty-one percent of respondents work in practices that have a website. When asked if they use social media to communicate with patients, only 29% of dentists responded positively. There was a statistical significant association between the use of social media and practice location. The majority of the dentists from urban areas (78.6%) reported that they use social media for patient communication compared to dentists practising in rural locations (*X*^2^ = 10.383, *p*=0.006).

Regarding the electronic submission of dental benefits and ACC claims forms, 40% of respondents did not use this procedure, compared to 28% who used it routinely. Fourteen percent noted occasional electronic submission of these forms. The remaining 18% of the respondents reported that they did not have dental benefit patients and, as such, did not need this facility.

### 3.7. Innovations

The collected data indicated that 64% of respondents (*n*=121) owned an intraoral camera, with 43% (*n*=82) using it on a routine basis and 17% (*n*=32) using it “occasionally.” The types of camera used are described in Table 3.

Table 4 describes the recent innovations and techniques used by dental practitioners in New Zealand. The majority of respondents indicated more than one option for recent innovation and techniques.

Regarding the use lasers, the results are presented in Table 5.

Concerning the concept of chair-side indirect restorations, CAD-CAM restorations were being placed by 32% of respondents, and air abrasion tooth preparation was being used by 31% of respondents.

### 3.8. Equipment

The most frequently used style of chair-side equipment was “over the patient” (52%) compared to 36% using a “cart.” The remainder of respondents used either a combination or a different method entirely. LED light-curing units were used by 91% of respondents and halogen by only 6%. When asked if respondents check the output of their light-curing devices, 62% responded yes, with the most common timeframe being every 6 months (23% of those who responded yes). The mean number of high-speed handpieces per surgery was 6 (SD 5.1). In comparison, the mean number of red-ring/speed increasing handpieces was 2 (SD 2.09).

### 3.9. Preventive Dentistry

Topical fluorides were used by 91% of respondents. The most widely used topical treatment was practice-based gels (63%). Forty-five percent of respondents prescribed fluoride rinses for home use, and 27% prescribed home-use gels. The majority of dentists reported using fissure sealants on an occasional basis (61%), with the remainder using them either routinely (28%) or not at all (8%). Antibiotic prophylaxis for the prevention of infective endocarditis was used by a number of respondents, as summarised in Table 6.

A total of 98.2% of respondents believed that the prevention of dental disease contributes to improvements in general health and well-being.

### 3.10. Infection Control

The type of gloves commonly used by respondents is summarised in Table 7.

When asked on average how many times they changed their gloves during the course of a thirty-minute appointment, 40% typically wore only one pair. Some dentists changed gloves once (17%), twice (28%), or more than twice (13%).

Regarding three-in-one syringes, 31% of respondents used disposable tips. Of the remaining respondents, 65% used reusable tips and were confident that tips were adequately sterilised between uses.

Concerning what was done with light-curing units light guides between patients, 33% of respondents used a disposable sleeve that was replaced, and 28% used a disinfecting wipe. In some cases, multiple answers were given, including 17% using a combination of the above.

Relating to infection control and clinical governance, DCNZ inspections of dental practices are currently the main mechanism in place. Seventy-one percent of respondents felt that these were beneficial to the safety of patients. Other perceived benefits are summarised in Table 8. The majority of respondents selected 2 or more options.

### 3.11. Health of Dentists

Surveyed dentists experienced a number of different illnesses causing absence from clinical practice. The predominant illness leading to absence was cold/flu (41%), followed by gastrointestinal upset (10%), trauma (7%), and headaches/migraines (7%). Neck and back problems caused absences in only 5% of the respondents. In addition, 4.8% of the respondents indicated that they were absent from work due to stress and depression.

## 4. Discussion

This questionnaire study was designed to investigate the demographic and practice arrangements of a random sample of general dentists in New Zealand. It was considered timely and relevant due to what has been reported in other countries in which this topic had been investigated [6, 9]. The practising habits of dentists are influenced by numerous factors, including the country of graduation, postgraduate training, CPD, and other courses [10]. Therefore, comparing the findings from this study with what has previously been seen in Australia and the United Kingdom was also of interest. In the past, the use of a postal questionnaire has been an effective way of investigating demographic details about the profession. The response rate of 58% obtained in this study is close to the average seen across similar studies, with the length of the questionnaire, incentives, and subject matter influencing response rates [11].

The proportion of respondents who were male was 63.5%. Comparably, in a survey which was conducted to investigate the Australian dental practitioner workforce in 2012, 63.5% of the respondents were male. However, the proportion of female dentists increased to 36.5% in 2012 from 35.2 in 2011 [12]. Similarly, the UK-based study had a 67% male response rate in 2008 and a 73% male response rate in 2000 [6, 8]. This suggests a continuation of the trend that dentistry is becoming less male-dominated and is a popular choice of profession for both sexes across the globe.

The larger proportion of practices in city/town centres was consistent with previous findings and is almost certainly due to larger population numbers in city/town centres, therefore, warranting a larger patient-base and treatment need [6]. A similar result was reported for an Australian study where the majority of dentists practiced in major cities (76%) with only 0.9% of the dentists working in remote areas [13, 14]. In addition, major cities had the highest number per 100,000 population of practising dentists (63.1%) and remote areas had the lowest (25.7%) [14].

The majority of patients treated in general practice in New Zealand appear to be fee-paying private patients (82%). This is in agreement with Australia where the majority of the dental care is provided in the private practice sector (85%) and most private patients must pay for their dental care, either directly or through individually purchased private dental insurance [13, 15]. However, this is in stark contrast to the UK, where 57% of patients seen were treated under the auspices of the National Health Service (NHS), where free and subsidised care is both readily provided and available [6]. The 2009 NZ Oral Health Survey highlighted that there is still a high level of untreated decay in adult New Zealanders. Only one in two adults admitted to visiting the dentist on a yearly basis [2]. This is a similar attendance rate for the UK where free and subsidised care is readily available. In Australia, those in lower household income groups had higher rates of avoiding or delaying a visit to a dentist (42.1%) due to cost than those in higher income groups (16.8%). In addition, 44.9% adults aged 25–44 years avoided or delayed visiting a dentist due to cost [14]. Surprisingly, people who were eligible for public dental care avoided visiting a dentist due to cost (37.9%) than those who were not eligible (29.4%) [14]. This raises the question of why the population is avoiding dental care and whether cost is in fact the largest barrier for patients when considering attending the dentist. This is an area, which needs further investigation.

The New Zealand Dental Council has set a minimum of 50 hours, verifiable continuing professional development (CPD) hours per recertification cycle of five years for dentists to maintain and develop their competence. Examples of verifiable CPDs include attending conferences, courses and workshops, web based study, postgraduate study, peer contact activities and presentation at oral health seminars. The Dental board of Australia has set a minimum of 60 hours of CPD activities over a three-year CPD cycle. With the majority of respondents (71%) in this survey attending five or more CPD courses per year, it would appear that dentists are putting great importance on developing their skills and keeping up to date with current evidence-based practice, innovations and technologies. This is slightly higher than 63% of respondents who were found to attend five or more courses per year in a UK-based study [6]. Similarly, a survey conducted in Victoria, Australia showed that dentists reported attending an average of 30.9 hours of certified clinical CPD over a 12 month period with the most popular popular method of CPD being attending courses (92%), followed by reading dental journals (85.1%), discussion with colleagues (68.7%) and attending dental conferences (67.9%) [16]. This would be in line with the findings or Mercer et al. who reported 88% of respondents in their study felt that CPD leads to a rise in morale and improvement in patient care [17]. In addition to this, the need to update clinical skills and integrate new developments into patient care is an accepted part of professional practice [10]. It would be interesting to investigate further in which country the respondents trained in, as differing CPD requirements between countries have a significant impact on the techniques used in general practice. This will be explored in the second part of this series.

Respondents using local anaesthesia as the most commonly employed method of pain control are consistent with the findings of previous studies [6]. Articaine was the most popular local anaesthetic of choice for 77% of respondents. There is evidence to support this choice, with articaine found to be more effective than lidocaine in providing anaesthetic success in the first molar region and both drugs having similar adverse effects profile [18].

There is a certain amount of competition between practices to market themselves in a way that attracts more patients and differentiates them from their competitors. Staying up to date with innovations may play a part in this, as in an age where technology is becoming an increasingly dominant part of everyday life, patients may be looking for similar trends in dental practice. It is therefore reasonable to assume that patients would expect practices to up-skill and keep up to date with new advances in materials and technologies.

Participants were asked about their use of recent technological advances in the profession and some pre-existing but still developing innovations and techniques, so that comparisons could be drawn with previous Australia and UK-based studies. It would appear that the use of computers has become an integral part of general dental practice in NZ, with 91% of respondents using a computer-based patient management system. According to the Australian Dental association, 90% of dental practices operate computer-based systems for billing, scheduling, and record keeping. In addition, 60% dentists use a computer at chair side, and the use of clinical computers has increased greatly over the last decade [19]. The majority of respondents (95%) had an Internet connection, and 81% of the respondents stated that their practice had a website that is similar to the findings in the recent UK-based study where 77.9% of respondents stated that their practice had a website, and 56.8% used the Internet to communicate with patients [20]. This should come as no surprise as New Zealand is frequently a first adopter of new technologies and would appear to be in line with current societal norms. Although a higher proportion of respondents noted having access to an intraoral camera compared to the UK, the regular use of this equipment was similar with what has been reported previously in the UK (43% vs. 48%) [6]. The use of digital radiography was common among respondents (82%), which is much higher than the 28% reported in the UK study, suggesting a move away from traditional analogue films [6]. This could be due to reasons of time management and efficiency of digital systems. Amongst other known advantages of digital radiography, there are overall reduction in dose, immediate availability of the image, and image enhancement functions [21].

A major difference between general dental practices in NZ compared to other jurisdictions is the routine use of antibiotic prophylaxis for the prevention of infective endocarditis. The evidence base for this continues to change and be updated and varies between countries, partly due to varying patient susceptibilities. Guidelines for the use of antibiotic prophylaxis in patients at risk of infective endocarditis, and orthopaedic patients with prosthetic joint replacements, are available from the NZDA [22]. 57.6% of respondents from this study chose to prescribe antibiotic prophylaxis in both of these scenarios, highlighting that it is still relatively commonplace and variable among general dentists in New Zealand. However, in Australia, antibiotic prophylaxis in not routinely recommended for patients with prosthetic joints who are undergoing dental treatment [23].

A large proportion of respondents (63.1%) are still using latex gloves rather than other latex-free alternatives, which is an increasing trend else where [6], despite the known association between latex and occupational contact dermatitis [24]. One previous New Zealand-based study noted as high as 40% of respondents affected by symptoms of contact dermatoses over the course of their practising careers, which may be explained by the higher usage of latex gloves [25].

Dental practice is considered as a stressful health care profession [26]. In general, dentists experience more physical and mental ill health issues compared to other professions due to patient behaviours and economic pressures [27, 28]. However, our study showed only 4.8% of the dentists in NZ reported being affected by stress causing absence from work. The majority of dentists were absent due to other general illnesses such as cold/flu, headaches, and gastrointestinal upsets. Future studies should investigate whether stress will become a major cause of work absences for dentists in the future.

The findings of this study highlight that dentistry is an ever-changing profession, with New Zealand dentists continuing to develop, learn, and embrace advancements in technologies, techniques, and materials that are constantly being introduced. As the profession strives to improve and advance, it is important that general dental practice in New Zealand continues to supply high-quality evidence-based treatment for the general public.

## 5. Conclusion

It is important to acknowledge that studies such as this one have a number of limitations. Data obtained in the current study are related to dental practitioners in New Zealand who responded to the study; however, findings and conclusions reported here can be applicable to other countries with similar practising arrangements. Sampling was done proportionally to the number of registered dentists in each of the 14 regions in New Zealand; however, the proportion of responses received from each region was variable and beyond the control of the study. Therefore, the response was weighted according to the responses for each region. The findings of this survey provide an updated and useful insight into dental clinical practice in New Zealand.

## Figures and Tables

**Table 1:** Proportional weight calculation with respect to New Zealand regions.

	Unweighted		*W*p^*∗*^	Weighted	
	Frequency	Percent	(*P*_*i*_/*P*_total_)/(*R*_*i*_/*R*_total_)	Frequency	Percent
Auckland	52	27.7	1.15	60	30.5
Bay of Plenty	14	7.4	0.85	12	6.1
Canterbury	21	11.2	1.22	26	13.1
Hawke's Bay	10	5.3	0.62	6	3.2
Manawatu-Wanganui	9	4.8	1.14	10	5.2
Nelson-Tasman	8	4.3	0.78	6	3.2
Northland	8	4.3	0.68	5	2.8
Otago	17	9.0	1.01	17	8.8
Southland	4	2.1	0.71	3	1.4
Taranaki	6	3.2	0.75	5	2.3
Waikato	13	6.9	1.57	20	10.4
Wellington	26	13.8	1.0	26	13.1
Total	188	100	—	196	100.0

^*∗*^ *W*p = proportional weight; *P*_i_ = total number of dentists in a particular region or sample; *P*_total_ = total dentist population in New Zealand; *R*_i_ = total number of dentists returned and met the inclusion criteria for the region; *R*_total_ = total number of dentists returned and met the inclusion criteria in New Zealand. *W*p > 1: sample under represented, *W*p < 1: sample over represented, and *W*p = 1: sample is proportional to total population.

**Table 2:** Availability of appointments for nonurgent care.

When is the first available appointment for nonurgent care in your practice?		
	Actual frequency (*n*=188)	Weighted percent (%)
The same day	41	23.0
Next day	21	12.4
Within five working days	78	40.5
Within three weeks	30	15.1
More than three weeks	9	3.7
More than one answer	9	5.3

**Table 3:** Types of camera used.

Camera type	Actual frequency (*n*=188)	Weighted percent (%)
Digital compact	39	21.4
Digital SLE	34	17.9
Video	17	9.5
More than one type of the above	15	7.8
Others	9	4.7
Not applicable	65	36.5
Did not respond	9	2.5

**Table 4:** Recent innovations and techniques.

Innovation/technique	Frequency	Weighted percent (%)
Air abrasion tooth preparation	62	30.8
CAD-CAM restorations	60	32.3
Diagnostic software	38	20.3
Digital X-rays/digital imaging	152	82.2
Guided tissue regeneration	9	4.5
Nickel-titanium endo-files	158	83.9
Zirconium based all ceramic bridgework	89	49.1
Fibre reinforced resin composite bridgework	66	36.2
Cone beam CT (CBCT) imaging	44	23.4
Tricalcium silicate	46	24.0
Mineral trioxide aggregare (MTA)	64	33.2
Laser hard and soft tissue	8	3.7

**Table 5:** Use of lasers.

Use of lasers	Actual frequency (*n*=188)	Weighted percent (%)
I do not own a laser and I do not like to	50	26.4
I do not own a laser but would like to	65	33.5
I own a laser and use it	61	33.6
I own a laser and do not use it	10	5.3
Did not respond	2	1.2

**Table 6:** Use of antibiotic prophylaxis in general dental practice.

For which patients do you routinely prescribe antibiotic prophylaxis?		
	Actual frequency (*n*=188)	Weighted percent (%)
(a) Patients at the risk of infective endocarditis	63	33.5
(b) Patients who have undergone prosthetic joint and replacement surgery	0	—
Both a and b	109	57.6
Both *a*, *b*, and others^*∗*^	13	6.91
Did not respond	3	1.9

^*∗*^Patients at the risk of infective endocarditis, usually ask GP for opinion, according to Heart Foundation recommendations and organ transplant.

**Table 7:** Type of gloves used by respondents.

Type of gloves	Actual frequency (*n*=188)	Weighted percent (%)
Powdered latex	30	15.7
Powdered latex-free	9	5.0
Powder-free latex	90	47.4
Powder-free latex-free	46	25.0
Combination of above	13	6.9

**Table 8:** Perceived benefits of DCNZ inspections.

Beneficiary of DCNZ inspections	Frequency	Weighted percent (%)
The dental team	108	57.7
Safety of patients	137	71.7
Achieving efficiency in the provision of dental care	66	34.9
Clinical outcomes	53	27.4
Patient trust and confidence in dental care	101	52.7
No one	19	10.2
Others	14	7.14

## Data Availability

The frequency and percentage data used to support the findings of this study are included within the article.
